# The Potential Influence of the Bacterial Microbiome on the Development and Progression of ADHD

**DOI:** 10.3390/nu11112805

**Published:** 2019-11-17

**Authors:** Stephanie Bull-Larsen, M. Hasan Mohajeri

**Affiliations:** Institute of Anatomy, Department of medicine, University of Zurich, Winterthurerstrasse 190, 8057 Zürich, Switzerland; stephanie.bull-larsen@uzh.ch

**Keywords:** microbiome, microbiota-gut-brain axis, ADHD, attention-deficit-hyperactive-disorder

## Abstract

The latest research cumulates staggering information about the correlation between the microbiota-gut-brain axis and neurodevelopmental disorders. This review aims to shed light on the potential influence of the microbiome on the development of the most prevalent neurodevelopmental disease, attention-deficit-hyperactive disorder (ADHD). As the etiology and pathophysiology of ADHD are still unclear, finding viable biomarkers and effective treatment still represent a challenge. Therefore, we focused on factors that have been associated with a higher risk of developing ADHD, while simultaneously influencing the microbial composition. We reviewed the effect of a differing microbial makeup on neurotransmitter concentrations important in the pathophysiology of ADHD. Additionally, we deduced factors that correlate with a high prevalence of ADHD, while simultaneously affecting the gut microbiome, such as emergency c-sections, and premature birth as the former leads to a decrease of the gut microbial diversity and the latter causes neuroprotective *Lactobacillus* levels to be reduced. Also, we assessed nutritional influences, such as breastfeeding, ingestion of short-chain fatty acids (SCFAs) and polyunsaturated fatty acids (PUFAs) on the host′s microbiome and development of ADHD. Finally, we discussed the potential significance of *Bifidobacterium* as a biomarker for ADHD, the importance of preventing premature birth as prophylaxis and nutrition as a prospective therapeutic measurement against ADHD.

## 1. Introduction

The microbiota-gut-brain axis is a bidirectional communication pathway between the microbiota, gut and central nervous system (CNS). It has been estimated that over 10^14^ microorganisms, which include bacteria, archaea, and eukaryota, reside in the gastrointestinal tract (GI-tract) [1]. According to the latest study, this results in an approximately equal number of microbial compared to human cells in an individual [2]. The microorganisms residing in the GI-tract play an important role in protecting humans from potential GI pathogens [3], and also exert neuroactive properties which explains why this ecosystem does not only influence the gut, but also the brain. Research shows the great importance of a healthy microbial composition in the gut at an early stage in life (2–3 years of age), a period also characterized by intense neurodevelopment in humans. Several reports conclude that early gut dysbiosis can influence the neurodevelopment in the short run and may also lead to mental health issues later in life [4,5].

Research highlights this risk, as gut dysbiosis in child or adulthood has not only been associated with various diseases, such as irritable bowel syndrome [6] or obesity [7], but also with psychiatric disorders as, for example, depression [8], Parkinson′s disease (PD) [9], schizophrenia [10], autism spectrum disorder (AS) [11], and lastly, attention-deficite-hyperactive-disorder (ADHD) [12].

ADHD is an early onset neurodevelopmental disease that, according to the fifth edition of Diagnostic and Statistical Manual (DSM-V), can be characterized into different representations: Hyperactivity and/or impulsivity, inattentiveness or all combined [13]. The worldwide prevalence of ADHD in children under the age of 18 ranges from 5.3% [14] to 7.2% [15], making it the most frequent neurobehavioral diagnosis in children. Interestingly, varying prevalence levels are reported in different geographies, which are primarily due to different characteristics of methods employed for ADHD diagnosis rather than geographic variations [14]. Nonetheless, 30–60% of the children continue to show symptoms into adulthood and thus, 1–6% of the population develop adult ADHD [16]. This is predominantly represented by the inattentive type [17].

This literature review attempts to identify and discuss factors that may influence the microbiome, and thus, could be associated with the development or progression of ADHD. Thereby we concentrate solely on the influence of bacteria rather than archaea and eukaryota. Furthermore, we evaluate the biochemical changes in ADHD patients and to what extent these can be related to microbial alterations in the gut. Finally, we reconfirm known biomarkers and deduce possible new ones for the diagnosis of ADHD and conclude what factors worsen or alleviate the development and progression of ADHD as this might lead to potential intervention methods of the neurodevelopmental disorder.

## 2. Materials and Methods

The key research question of this literature review is: What factors may influence the microbiome and could be associated with the development and/or progression of ADHD? The databases Pubmed and Scopus were searched until the 1 July 2019 with the following MeSH and search terms: “Microbiome”, “microbiota”, “gut-brain axis”, “microbiota-gut-brain-axis”, “ADHD”. Most of the research papers included in this review were published between 2010 and 2019.

As our primary focus was on bacteria, we excluded studies that concentrated on archaea and eukaryota. The incorporated studies had to fulfill all of the following inclusion criteria:

- Articles were directly related to the topic;
- ADHD patients were diagnosed by a medical expert;
- Publication in a peer-reviewed journal;
- Availability of the full-text publication;
- Studies were written in English.

A total of 208 citations were included in this article.

## 3. Evidence Linking Microbiota to ADHD

### 3.1. Microbiome

The influence of the microbiome on the ADHD pathophysiology is being intensively researched. The microbiota consists of the different microorganisms [18], and the microbiome describes the entire genome of the microbiota [18]. The primary functions of the microbiota include: (i) Protecting the host organism against pathogens by increasing the mucine production, and thus, stabilizing the gut-blood barrier; (ii) support of the immune system [19]; (iii) the production of vitamins [20]; and (iv) short-chain fatty acids (SCFAs), whereby the latter are products of microbial catabolism of indigestible carbohydrates [21]. Throughout the GI-tract, the composition and density of microbes changes, increasing from 10^2^ cells per gram of content in the stomach to 10^8^ cells per gram in the cecum [22]. Additionally, up to 1000 different bacterial species have been found to inhabit the GI-tract of humans [23]. Thereby the composition in species of the microbiome can be influenced by genetics [24], geography [25], disease, medication [26], and age [27].

The GI-microbiota goes through a physiological change from its prenatal period until the age of three [27]. For a long time, it has been thought that the intrauterine environment is sterile and that the first bacterial colonization of the newborn happens during delivery [28]. However, numerous studies have shown that bacteria exist in the placenta, amniotic fluid [29,30,31], and meconium [32] indicating that the unique microbial composition in utero may already influence the development of the microbiome of the fetus before birth. Research demonstrates that the microbiome of the placenta is low in richness and diversity and is predominantly colonized by the phyla Proteobacteria and Bacteroidetes. The former is mostly represented by the spp. *Escherichia coli* and *Neisseria lactamica*, while Bacteroidetes is dominated by *Bacteroides* spp. [31]. Other important phyla include Firmicutes, Fusobacteria and Tenericutes [31], whereby the latter includes genera, such as *Mycoplasma* and *Ureaplasma* [33].

**Figure 1:**
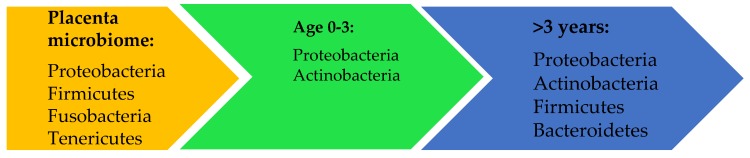
The most prevalent bacterial phyla in utero and in the GI-tract of humans. This figure represents the dynamic and development of the composition of the microbiome from fetuses in utero until the age of three years, at which point the microbiome gains its stability and consists of mostly four phyla: Proteobacteria, Actinobacteria, Firmicutes, and Bacteroides.

The colonization of the gut in the postnatal period is sensitive to environmental factors. Nonetheless, the normal composition of the microbiome in a newborn is low in diversity and shows dominance in Proteobacteria and Actinobacteria [34]. More specifically, Proteobacteria shows its peak at birth, whereas Actinobacteria increases and dominates at the age of four months [35]. At this point, Proteobacteria is still mostly represented by *Escherichia coli* and Actinobacteria by the genus *Bifidobacterium longum* [35]. As seen in Figure 1, at the age of three and onwards, the microbiome stabilizes to four major phyla: Firmicutes, Bacteroidetes, Actinobacteria, and Proteobacteria, which normally cover more than 90% of the total bacterial population in a human body [36].

### 3.2. Gut-Brain Axis

The gut-brain axis describes the bidirectional communication between the microbes, enteric nervous system and the CNS [37]. So far, there are three known means of communication between these compartments: Neural, immune, and endocrine [4,38].

The neural pathway describes the hypothalamic-pituitary-adrenal axis (HPA axis), which is the most important efferent stress pathway. It is of great importance to understand to what extent the HPA axis plays a role in the pathogenesis of ADHD, as it influences pathways in the body that are often deviating in ADHD patients [39,40] as for example: Circadian rhythm [41], sleep [42], and emotions [43]. The stimulation of the HPA axis by stress or pro-inflammatory cytokines results in a release of corticotropin-releasing factor (CRF) from the hypothalamus, as well as adrenocorticotropic hormone (ACTH) from the pituitary gland, finally resulting in the secretion of cortisol from the suprarenal (adrenal) glands [38].

One study using 69 healthy children and 123 children with ADHD observed an increase in salivatory cortisol in ADHD patients after waking up in the morning [44]. The effect of stressors was studied in one paper showing that after being exposed to stress children with ADHD of combined type (high levels of hyperactivity and impulsivity) have decreased salivary cortisol levels in comparison to other ADHD patients [45]. In contrast, adult ADHD patients with an inattentive type showed higher levels of cortisol in comparison to the combined types, which showed normal levels of cortisol [46]. Finally, Lackschewitz et al. discovered that adults with ADHD who undergo a stress-inducing exam show a trend towards reduced cortisol levels [47]. These reports portray the association between altered cortisol levels and different types of ADHD. However, the heterogeneity of the results can be explained by various stressors on a differing target group all acting as confounders. Thus, only future studies using the same stressor, examining similar and large patient groups will allow drawing further reliable conclusions.

The neuroimmune communication pathway describes how intestinal microbes influence the function and maturation of immune cells in the CNS, whereby microglia cells play an important role [48]. These cells are activated, as well as produced, by pro-inflammatory cytokines, and are important regulators for autoimmunity, neuroinflammation, and neurogenesis [49]. Germ-free (GF) mice showed defects in microglia activation, which in turn lead to a deficient innate immune response when exposed to pathogenic bacteria [50]. The same study showed the immense effect the microbiome has on microglial cells, as introducing microbiota into GF mice resulted in restored microglial functions. Reversely, eradicating various bacteria in specific pathogen-free (SPF) mice resulted in microglial cells maturing less rapidly [50]. As neuroinflammation plays an important part in the pathophysiology of ADHD, the proper activation and maturation of microglia in ADHD patients have to be thoroughly investigated in order to determine if it has a pathogenic influence.

The enteric nervous system communicates with the brain mainly through the parasympathetic vagus nerve [51], and partially though the sympathetic spinal cord pathway [52]. Furthermore, the vagus nerve predominantly consists of afferent nerve fibers with a ratio of 9:1 to efferent fibers [53]. Even though a definite conclusion cannot be made, various studies have demonstrated that the autonomic nervous system of ADHD patients shows alterations. A study testing 19 children with ADHD showed that the patients had an underactive parasympathetic and an overactive sympathetic nervous system [54]. Another study comparing 32 ADHD patients to 34 healthy controls registered under-aroused parasympathetic nervous systems, while the sympathetic part did not show any difference between the groups [55].

It has become clear that all three ways of communication between the microbiome, gut, and CNS could play an important role in the pathophysiology of ADHD. The neural communication over the HPA axis shows abnormalities in ADHD patients. Additionally, studies detected that microbes influence the function of pro-inflammatory microglia, a key finding, as neuroinflammation in ADHD patients is commonly found. Finally, the autonomic nervous system shows aberrations as the main research results show an under-arousal of the parasympathetic nervous system.

### 3.3. Etiology of ADHD and the Genetic and Environmental Influences

As the exact pathophysiology of ADHD is still unclear, its causes are still being researched. Nevertheless, it has been established that there is an interplay between genes and the environment resulting in a complex etiology. Genetic predisposition plays an important part in the pathophysiology of ADHD as children from parents that have been diagnosed with ADHD have a 50% higher chance to be diagnosed with the same disorder [56]. Similarly, twin studies have shown a high heritability, as especially for inattentive and combined types an inheritance of 71–90% could be discovered [56,57]. On the other hand, one study showed, that 20–30% of the risk of developing ADHD is due to environmental factors [58]. These include perinatal maternal smoking, stress, mineral and micronutrient deficiencies and premature birth [59]. Additionally, research showed that 10–40% of the variance inheritance of ADHD could be caused due to the environment highlighting the interplay of genetic and environmental risk factors [60]. Due to these complex interactions, it is believed that ADHD can be manifested with highly heterogenous symptoms depending on the exact pathway and etiology involved [61].

Research shows that the dysfunction of monoaminergic neurotransmitters, including noradrenaline (NE), serotonin (5-HT) and dopamine (DA), plays an important role in the pathophysiology of ADHD [62].

#### 3.3.1. Dopamine

**Figure 2:**
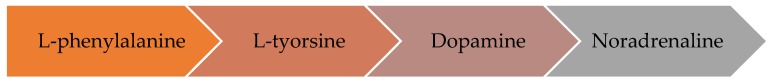
The synthesis pathway from L-phenylalanine to noradrenaline including all its intermediary products. Dopamine acts as an important metabolite for the emotional response and reward system [64].

DA is a catecholamine that acts both as a hormone and neurotransmitter (NT). It is a product of the essential amino acid L-phenylalanine, which must be provided in our diet. As seen in Figure 2, this is then turned into L-tyrosine, and finally into DA and NE [63].

The dopamine hypothesis links ADHD to alterations in dopamine metabolism. The hypothesis describes the increased expression of presynaptic dopamine transports (DAT) in ADHD patients leading to an increased dopamine transporter density (DTD), and finally results in a decreased level of the bioavailable NT [65]. The dopamine hypothesis gained attention due to the way methylphenidate (MPH) and amphetamines (AMP), the most commonly used pharmacotherapies to treat ADHD, interact with the DA and NE metabolism. MPH and AMP exert a stimulatory effect in ADHD as they inhibit the reuptake of NE and DA by blocking the metabolizing enzyme, monoamine oxidase (MAO), thereby increasing the concentration of the two monoamines in the synaptic cleft. One differentiates between MAO-A and MAO-B as the former is mostly expressed in the liver and GI-tract and the latter in blood platelets [66]. Nevertheless, both are manifested in the CNS and are able to break down DA [66]. Furthermore, amphetamines have the ability to release NTs from the presynaptic neuron, which additionally increases the monoaminergic concentration in the synapse [67].

Moreover, recent research shows that not only the metabolization, but also the production of DA plays an important role in the pathophysiology of ADHD. One of the influencers on the production of NTs seems to be the microbiome in the GI-tract [68]. Bacteria, such as the genus Bifidobacterium belonging to the phylum Actinobacteria potentially influence the levels of available DA in the body by encoding cyclohexadienyl dehydratase (CDT) [69]. This enzyme is important for the synthesis of the essential amino acid phenylalanine [69], which acts as a precursor of the amino acid tyrosine, which in turn is metabolized into DA and lastly to NE [70]. Aarts et al. found an increase in Bifidobacterium in ADHD patients, and thus, higher levels of CDT. By analyzing BOLD responses of the ventral striatal using fMRI measurements they deduced a negative correlation between the abundance of CDT and reward anticipation [69], a key symptom in ADHD [71], and target of DA [72]. Finally, this study concluded that high levels of phenylalanine might be a risk factor for abnormal dopamine signaling and could lead to a reduced reward response [69]. Although another study supports the findings by Aarts et al. [73], the correlation still appears to be inconsistent as two older studies found a decreased level of phenylalanine in ADHD patients [74,75], even if data of source [74] are not statistically significant. Finally, a more recent study found no correlation between phenylalanine levels and ADHD [76]. A summary of these finfings is given in Table 1.

#### 3.3.2. Tryptophan and Serotonin

Upon intestinal absorption into the bloodstream, the essential amino acid tryptophan can cross the blood-brain barrier (BBB). Thereby tryptophan can act as the precursor of the neurotransmitter 5-HT, which plays an important part in the microbiome-gut-brain axis [77]. Although it is still unclear to what extent the microbiome influences the synthesis of 5-HT, it has been established that certain strains of bacteria, such as *Streptococcus* spp., *Enterococcus* spp., and *Escheria* spp. are capable of producing this NT [78]. Most of the 5-HT is produced and stored in gastrointestinal cells and affects peristalsis, nausea, satiety and abdominal pain [79]. Meanwhile, in the brain, it influences other NTs, such as DA, Cholin (CH) and GABA, which influence memory and mood [80].

Banerjee et al. showed that 5-HT may have an influence on hyperactive and impulsive symptoms in ADHD [81]. Another study implied lower levels of 5-HT in the CNS of ADHD patients due to a decreased transport capacity of its precursor, tryptophan, into the brain [82]. Finally, one study showed that inflammation in the intestine affects 5-HT signaling pathways due to a decreased function and expression of the serotonin selective reuptake transporter (SERT) resulting in an increased level of 5-HT in the body [79]. However, it is important to remember that serotonin is not able to cross the BBB, and thus, the 5-HT pools in the CNS and the periphery do not directly interact with each other.

To demonstrate the importance of microbes on the 5-HT system, one study concluded that GF male mice have a 1.3 fold increased level of 5-HT in their hippocampus. This is an important finding as certain therapeutic medications of ADHD, such as escitalopram and lithium increase serotonin levels in a similar amount [83]. Thus, the composition and the modulation of the gut microbiota might become an interesting, future therapeutic intervention strategy.

Although the studies do not allow us to make a precise conclusion in what way bacterial-produced 5-HT influences ADHD, they do make it clear that it is one of the several catecholamines that play an important role in the pathophysiology of ADHD.

#### 3.3.3. Kynurenine Pathway

Although tryptophan is the key amino acid for the production of 5-HT, 90% of tryptophan is catabolized by the kynurenine pathway [84]. This process produces nicotinamide adenine dinucleotide (NAD) through the stimulation of inflammatory and glucocorticoid metabolites. The kynurenine pathway has received attention in regards to psychiatric diseases, such as depression and schizophrenia [80,85,86] as it uses most of the tryptophan, and thus, leaves a limiting amount of substrate for the synthesis of serotonin.

Intermediate products, such as kynurenine, kynurenic acid (KA), xanthurenic acid (XA) and quilonoic acid (QA) can influence the immune system and neurotransmission [87]. The three former metabolites have anti-inflammatory properties as KA inhibits the NMDA-gated ion channels [88], and XA interferes with the glutamatergic neurotransmission [89]. Also, these products decrease the amount of pro-inflammatory IFN gamma in comparison to the anti-inflammatory IL-10 [87]. In contrast, QA stimulates microglial cells and increases the ratio of IFN gamma/IL-10 [87], resulting in pro-inflammatory effects [90]. Although KA shows neuroprotective properties, human and animal studies show that high levels of KA are associated with cognitive abnormalities, such as attention and memory issues typically associated with psychiatric disease [91,92].

Studies regarding levels of tryptophan and metabolites of the kynurenine pathway show inconclusive results. A Norwegian study, using 133 adult ADHD patients and 133, did not find that the ADHD group had lower levels of tryptophan and neuroprotective KA and XA [86]. These data were confirmed by another study testing ADHD children, which exhibited lower KA and XA levels [93]. These researchers, however, recorded higher levels of tryptophan in ADHD subjects [93]. These data do suggest an association between low levels of KA and XA in ADHD, but as there are still too few studies on this topic, it is difficult to deduce a definitive connection between tryptophan, its metabolites, and ADHD.

The various steps of the kynurenine pathway are dependent on coenzymes, such as the activated form of vitamin B6, pyridoxal 5′-phosphate (PLP). One study found an inverse correlation between serum levels of vitamin B6 and ADHD including its symptom severity [94]. Similarly, Aarsland et al. also observed a decrease in vitamin B6 in their patient group. Other data suggested that vitamin B6 metabolism plays a key part in the pathophysiology of ADHD, as vitamin B6 dependent enzymes show severe abnormalities in the ADHD test group [95]. Thus, lower levels of intermediate metabolites could be related to a deficiency of enzyme substrate. This data supports the importance of optimal coverage of ADHD patients with vitamin B6. The microbiome could play a potentially important role, as bacteria in the large intestine produce this vitamin [96]. As the correlation between levels of vitamin B6 and ADHD are relatively new, future studies are warranted to asses to what extent the microbiome can influence vitamin B6 levels on a therapeutic level.

#### 3.3.4. Gut Dysbiosis and Immunology

High variability in gut flora prevents the growth of pathogenic bacteria, and thus, stops gut dysbiosis [97]. The term dysbiosis describes a microbial imbalance in which there is a shift from protective to pathogenic microbes in the GI-tract [98]. This can lead to a growing GI-permeability which leads to an increase in migration of pathogenic microbes and translocation of their metabolites into the systemic circulation potentially resulting in systemic inflammation [99]. This can, in turn, decrease the permeability of the BBB, which can lead to inflammation of brain parenchyma [100]. Severe dysbiosis has been associated with chronic inflammatory intestinal disorders and psychiatric illnesses, such as schizophrenia, anxiety, depression [98], and ADHD [90,101]. A systematic review supports the latter findings concluding that patients with ADHD have increased levels of inflammatory cytokines [102]. Similarly, Verlaet et al. also detected increased levels of pro-inflammatory cytokines (IFN gamma and IL-6) in the serum of ADHD patients [101].

An imbalance of pro-inflammatory cytokines can also lead to allergic disease [103], and a positive correlation between ADHD and allergies has been shown in different cohorts [104,105,106]. Additionally, research has shown an association between an altered gut microbial composition and the tendency to suffer from the allergic disease [107].

An important pro-inflammatory cytokine is interleukin (IL-6). This has been inversely associated with the bacterium *Dialister* spp. [108]. *Dialister* spp. is shown to correlate with an altered temperament and impulsiveness in toddlers positively. These commonly found ADHD symptoms were measured using the Early Childhood Behavior Questionnaire (ECBQ), which measures extroversion, activity levels and feelings of high-intensity pleasure [109]. Furthermore, a review evaluating multiple studies concluded an increase in pro-inflammatory metabolites, such as IL-6 and IL-1 in patients with ADHD [110]. Nonetheless, one study showed that ADHD patients had significantly lower levels of *Dialister* spp. in comparison to healthy controls (HC), hinting towards decreased feelings of activity and lower levels of intense pleasure, and finally higher levels of IL-6 [111].

Although the association between *Dialister* spp. and feelings of pleasure are new findings; and it is important to note that pro-inflammatory interleukin levels are increased in ADHD patients. As high levels of pro-inflammatory interleukins can be linked to neurological inflammation that can lead to a decrease of cortical volume and altered behavior [110,112], reducing the activity of these pro-inflammatory cytokines could represent a vital prophylaxis strategy in ADHD management.

Patients with th2-mediated atopic disorders, such as eczema, asthma and allergic rhinitis have a 30–50% higher chance of developing ADHD [113]. Eczema is an inflammatory skin disease and is the most prevalent chronic condition in early childhood [114]. Children suffering from atopic dermatitis (eczema) have a 50% likelihood of developing asthma and allergic rhinitis, exhibiting airway inflammation and clear nasal discharge, respectively [115]. Th2-cytokines are important for eosinophilic recruitment and the production of IgE by B-lymphocytes. All of these processes are associated with allergies and inflammation of the skin (e.g., eczema) [116,117] as they activate the production of pro-inflammatory cytokines, such as IL-6, IL-1beta, TNF-alpha and IL-8 [103]. Studies have shown that these atopic diseases are associated with a low level of *Faecalbacteria* spp. In the gut [118]. This species is known to have anti-inflammatory effects on the organisms [119,120]. As explained above, patients with ADHD seem to exhibit higher levels of inflammatory markers which could potentially support the hypothesis that low levels of *Feacalbacteria* spp. Cause an increase of inflammation which affects the development of the brain, and finally the pathogenesis of ADHD.

## 4. Results

### 4.1. Obstetric Mode of Delivery: Vaginal Birth vs. Caesarean Section (C-Section)

As infants delivered by vaginal birth move through the birth canal, they get colonized by their mother′s vaginal microbiota, and thus, adopted a resembling gut microbiome. In contrast, infants born via c-section are colonized by the microbiota of their mother′s skin. Therefore, the delivery mode affects the composition of the gut microbiota in infants [121].

Results of various studies showed that in comparison to vaginally born infants, babies delivered by c-section had a decreased gut microbiota diversity including lower levels of *Bifidobacterium* spp. and Bacteroidetes, but increased levels *Clostridium difficile* [122] up until the age of two years [123].

Several research groups studied the correlation between c-section delivery and ADHD (see Table 2). An animal study showed a correlation between offspring born via c-section and altered dopamine metabolism throughout development [124]. It is important to note that these results might have been confounded by indication, which means that the altered dopamine response might be due to triggers that lead to a c-section [125]. In contrast to the above findings, two previous case-control studies found no significant correlation between c-sections and ADHD [126,127] in humans. A systematic review by Curran et al. initially showed a slight increase in the prevalence of ADHD in children born via c-section [128]. This correlation was challenged in their later study due to confounders, such as not differentiating between elective and emergency c-sections [129]. The only correlation that still seemed to be consistent was an increased prevalence of ADHD in children born via emergency c-sections. Confirmative data were obtained in a prospective cohort study using 671,592 Danish children. They found a significantly increased chance of children developing ADHD (Hazard Ratio 1.21) for intrapartum c-sections, but no effect when born by an elective c-section [130]. In contrast, the Millenium UK cohort study testing 13,141 children found no correlation between ADHD and mode of delivery despite differentiating between emergency, planned and induced c-sections [131].

The reasons for finding a positive correlation between intrapartum c-sections and ADHD development cannot unequivocally be explained as multiple confounders, such as unobserved familial factors, birth weight or gestational age also directly influence the mode of delivery and ADHD. However, there is a strong indication that the microbiota plays a subordinate role in this correlation as Axelsson et al. discovered that exposure of the newborn to ruptured vs. non-ruptured membranes prior to c-section did not influence the correlation between c-section and ADHD development [130]. 

To conclude, the accumulative data show that the mode of delivery affects the composition of the gut microbiota. However, a clear correlation between c-section delivery and a higher chance of developing ADHD cannot be found as results depend on various confounders and the type of c-section, whereby intrapartum c-sections show a positive correlation with the development of ADHD in comparison to elective c-sections.

### 4.2. Stress of the Mother

A prospective follow up study, and a Dutch population-based cohort study concluded a correlation between prenatal maternal stress exposure and an increase in ADHD in their offspring [132,133]. This data was confirmed by a Canadian study enrolling 203 pregnant women exposed to stress. Sixty-two of them were exposed to severe prenatal stress (experienced physical or sexual abuse, or death of a close relative) and delivered children with more severe ADHD symptoms in comparison to the 48 mothers who experienced moderate stress (financial or marital troubles) [134].

An animal study using quantitative PCR determined that maternal stress significantly decreased one of the most abundant taxa in the maternal vaginal flora, *Lactobacillus* spp. [135]. Consequently, *Lactobacillus* spp. was also significantly decreased in the distal colon of the offspring of stress exposed mothers. Additionally, a review focusing on the immunomodulatory effects of *Lactobacillus* spp. shows that stress reduces the abundance of this species independent of the host being pregnant or not [136]. *Lactobacillus* spp. is important for the synthesis of acetylcholine, while together with *Bifidobacteria* spp. it is contributing to the production of the main inhibitory neurotransmitter GABA [137]. Alterations in the GABAergic system have been associated with neurodevelopmental diseases, such as autism spectrum disorder and ADHD. This system is especially susceptible to alterations during development as GABAergic neurons originate from a different part of the neural tube than GABA′s most important counterpart, the glutamatergic system. ADHD symptoms may be explained by the hypothesis that inhibitory functions of the cerebral cortex are reduced, leading to a reduction of filtering sensory influences, and finally having difficulties choosing the right behavioral reaction [138]. 

As described above, several studies have associated low levels of cerebral GABA concentrations with symptoms of ADHD [139,140,141]. Furthermore, a randomized controlled study showed that *Lactobacillus rhamnosus* also has a preventive effect as the administration of this bacterium in the first six months of life reduced the risk of ADHD and Asperger Syndrome (AS) [142]. The positive effects of this species may be due to the fact that *Lactobacillus rhamnosus* is, on the one hand, implicated in the development of tight junctions responsible for a strong gut barrier, and on the other hand, important for the immunoglobulin A and mucin production [143].

Various factors influence the development of ADHD, among which the neuroinhibitory neurotransmitter GABA seems to play a crucial role. However, to what extent low levels of *Lactobacillus* spp. and decreased concentrations of GABA are associated and how they precisely affect the development of ADHD remains unclear and has to be thoroughly investigated.

### 4.3. Preterm

Preterm babies that, thus, have gone through stressful situations similarly show lower levels of *Lactobacillus* spp. [144], and simultaneously have a significant increase in the prevalence of ADHD [58,145,146]. More specifically, Barrett et al. showed an increased abundance of *Proteobacteria* spp., while discovering undetectable levels of *Lactobacillus* spp. and *Bifidobacteria* spp. [144,147]. Chou et al. discovered that certain strains of *Lactobacillus* spp. show a protective trait towards the CNS. Preterm babies received strains of *Lactobacillus reuteri* and *Lactobacillus rhamnous* as probiotics for six weeks. This treatment resulted in a significantly reduced number of babies with neurological aberrations at one year of age in comparison to the group fed with *Lactobacillus acidophilus* and *Bifidobacterium infantis* [148].

It is widely known that preterm infants own an immature immune system as the innate and adaptive immune system has not developed fully. Due to their immature immune response and their usual extended hospital stay, infants are highly susceptible to nosocomial spread infections [149]. The increased number of infections may impair the neurodevelopment, and thus, might influence the development of the most common neurodevelopmental disorder ADHD. In addition to this, the weeks before term delivery (between 37 0/7 and 41 6/7) [150] represent an important stage in the neurodevelopment of the brain usually occurring in the protective womb of the mother [151,152]. Thus, preterm babies suffer from underdeveloped brain structures that in combination with postnatal complications, such as infections can lead to cell death of neurons, and finally lead to a decrease in the volume of specific areas of the brain [153].

As the prevalence of infection in premature newborns is high, the use of therapeutic antibiotics is similarly increased. Antibiotics have been associated with altering functions in the host′s brain [154], while simultaneously, they are notoriously known for reducing the diversity of the microbiota [155]. Nevertheless, the direct effects of a lower microbiota diversity on the neurodevelopment have still not been thoroughly researched on, and thus, a concrete correlation cannot be made.

To summarize, a preterm baby is exposed to increased levels of stress, may have underdeveloped brain structures and owns an immature immune system. All of these result in a higher susceptibility to infections, and finally may lead to increased exposure to therapeutic antibiotics. These factors influence the neurodevelopment either directly through inflammatory processes during infections or indirectly by changing the composition of the gut microbiome.

### 4.4. Breastfeeding vs. Formula Feeding

Studies have associated breastfeeding with a lower prevalence of ADHD [156]. In contrast, formula-fed newborns showed a strong correlation with ADHD diagnosis [157,158,159]. The nutritious breast milk not only contains human milk oligosaccharides acting as prebiotics important for establishing a healthy gut microbiome, but also consists of vitamins and antibodies [160]. The latter being important in the first couple of months for the maturation of the innate immune system of the newborn [161]. Additionally, breast milk is marked to have a rich fat content due to its high levels of long-chain fatty acids, which are said to have protective effects on the CNS and the development of ADHD [162,163]. The gut microbiota of breastfed infants is less diverse in comparison to formula-fed infants [164]. Importantly, various groups utilizing differing methods for microbiome analysis, such as 16S sequencing or cytogenic FISH technique discovered that in both groups, the most prevalent genus is *Bifidobacterium* [122,164,165].

A systematic review by Guaraldi et al. showed that bottle-fed infants have a higher number of *Escherichia coli*, *Clostridium difficile*, *Bacteroides* spp. and *Lactobacilli* [166]. As seen in Table 3, research papers demonstrated that increased levels of *Lactobacillus acidophilus* [122], *Streptococcus*, *Veillonella parvula* [164], and *Clostridium coccoides* [165], were found in formula-fed infants. Although *Bifidobacterium* is the most prevalent genus found in both groups, breastfed infants show more than double of *Bifidobacteria* cells in comparison to formula-fed infants [164]. *Bifidobacterium infantis* has protective properties against pathogens as it supports the barrier function of the mucosa and concurrently has anti-inflammatory properties, thus, promotes a healthy immunological response [167,168].

Although the effect on the microbial composition could be the main cause of developing ADHD when being formula-fed, one has to consider the fact that other ingredients in the formula may also act as important influences. One study found that there were more cases of ADHD in formula-fed infants in 2007, than in December 2011. During the latter, the neurotoxic chemical Bisphenol A (BPA) was significantly reduced in formula cans and baby bottles in comparison to the former, suggesting that BPA might be the actual trigger of the correlation [169].

In summary, studies show that breastfeeding correlates negatively with the risk of developing ADHD, whereas formula-feeding increases this risk. Nevertheless, despite the highly nutritious content of breast milk, the gut microbiota of breastfed infants seems less diverse, but still contains the same or higher amount of protective components than formula-fed infants. Thus, alteration of the microbiome composition could potentially be a reason for the positive correlation between formula-feeding and the risk of developing ADHD.

### 4.5. Short Chain Fatty Acids

SCFAs are products of polysaccharides which could not be properly digested by the human digestive system, and thus, are broken down by microbial fermentation. Bacteria, such as *Bacteroides* spp. and *Clostridiae* spp., are two of the most important microbes for the production of SCFAs [21]. SCFAs represent not only a major energy source for microorganisms, but also show neuroactive and anti-inflammatory effects on the host [170,171]. A study by MacFabe et al. demonstrated that when SCFAs, such as propionic acid are intracebreoventriculary (ICV), administered to rodents, they show biochemical alterations similar to individuals who from autism [172]. Besides, the same authors found that high levels of the SCFA worsened symptoms of autistic individuals [172].

As ADHD, similar to autism, is a neurodevelopmental disease, it seems likely that SCFAs may affect the development of ADHD. Research shows that SCFAs influence the immune system, and as discussed earlier, this can influence the CNS [50]. An animal study using mice showed that the microbiome could influence the levels of the brain-derived neurotrophic factor (BDNF) via SCFA production [173]. The neurotrophin BDNF is important for neurogenesis and has a positive effect on the survival of neurons meaning that the microbiome can indirectly influence neural functions via SCFA′s modulating effect on the BDNF production. The same study showed that GF mice whose BDNF levels had been decreased displayed problems with their working memory [173]. Confirmatory data were generated by Corominas-Roso et al. who showed in a human study that adults with ADHD have lower levels of BDNF compared to healthy controls [174]. Similarly, Akay et al. tested the effects of methylphenidate on BDNF levels on 50 drug-naïve ADHD boys and detected significantly increased BDNF levels in the serum and improved ADHD symptoms after eight weeks of methylphenidate treatment [175]. The same findings were found by an older study by Amiri et al. [176]. This is a direct confirmation of a potential link between the dopaminergic system, BDNF function, and ADHD. In contrast, another study enrolling 41 untreated ADHD and 107 control patients concluded that drug-naïve ADHD children had higher levels of BDNF in their plasma and that these levels are positively associated with the severity of inattentiveness [177].

Besides hypothesizing a compensatory mechanism in ADHD children, a potential reason for these differing results could be varying methodology as Akay et al. measured BDNF levels in the serum, known to have a higher BDNF concertation in comparison to the plasma [178].

In conclusion, SCFAs most probably affect the development of ADHD indirectly by influencing the production of BDNF.

### 4.6. Polyunsaturated Fatty Acids

Another regulator of BDNF seems to be omega-3 polyunsaturated fatty acids (PUFAs). PUFAs are long chains of carbon atoms characterized by a carboxyl group at one end and a methyl group at the other end. As they are unsaturated, they own one or more double bonds between the carbon atoms. Naturally, plant and fish oils, such as flaxseed or salmon have a high content of omega-3 PUFAs [179]. PUFAs play an important role in membrane fluidity, neuronal membranes, neurotransmission, and receptor function [180]. Furthermore, the omega-3 fatty acid, docosahexaenoic acid (DHA), is indispensable for cognition function throughout the lifespan [181]. Indeed, already intrauterine PUFA deficiencies lead to altered cognitive and attentive skills [182].

An animal study showed that omega-3 PUFAs did not only affect the levels of BDNF, but also of glial cell-derived neurotrophic factor (GDNF). The latter is especially important for the recovery of dopaminergic neurons in Parkinson′s disease (PD) as it promotes the survival of the dopamine system in the nigrostriatum. Hence, GDNF is shown to be neuroprotective and supporting dopaminergic neurons in PD models, and thus, could potentially be utilized as a therapy against neurodegenerative diseases, especially PD [183,184]. Furthermore, another study found that lower levels of omega-3 fatty acids were associated with lower levels of BDNF in the frontal cortex of rats [185], a part of the brain where various psychiatric illnesses, such as bipolar disease can be manifested [186]. Additionally, omega-3 PUFAs show antimicrobial effects as they increase levels of *Enterobacteria* and *Bifidobacteria*, which both strengthen intestinal permeability, reducing the risk of inflammation [187]. Finally, omega-3 PUFAs have the ability to stimulate macrophages that inhibit the activation of the NLRP3 inflammasome, and thus, decrease levels of the previously mentioned pro-inflammatory IL-1beta [188]. Nevertheless, it is important to note that an excess of omega-6 PUFAs benefits the development of endotoxemia leading to low-grade systematic inflammation, explaining why a low ratio of omega-6/omega-3 PUFAs should be targeted [189,190].

Human studies have discovered a negative correlation between patients with ADHD and levels of PUFAs. An Italian study examined the levels of PUFAs in the blood of 51 ADHD and 22 non-ADHD patients. PUFA levels in the blood of ADHD patients were significantly lower and correlated with behavioral symptoms, but were not associated with cognitive skills [191]. Similarly, a systematic review concluded that in all randomized control trials (RCT) analyzed (7 RCTs, n = 534), omega-3 PUFA supplementation led to an improvement in clinical ADHD symptoms. Furthermore, in three out of the seven RCT′s (n = 396), the omega-3 PUFA supplementation was associated with improvements in cognitive skills [192]. Due to these findings, questions of PUFAs being a potential therapeutic medication for ADHD patients seem to be warranted.

Moreover, a double-blind trial [193] assessed the effects of inducing the noradrenaline reuptake inhibitor (Atomoxetine) conventionally used to treat ADHD, to the patient and control group and PUFAs, such as eicosapentanoic acid (EPA) and DHA solely to the ADHD patients. The medication was given on a daily basis for four months to a total of 50 children. Although PUFAs improved ADHD symptoms, this experiment showed no clinically significant difference in the ADHD Conners Parent rating scale, questioning the overall therapeutic effect of PUFAs against ADHD, even if some beneficial effects are evident [193]. Supporting these results, a systematic review discussing results of 14 meta-analyses inducing PUFAs to ADHD children showed a very small effect size when parents and teachers rated children′s behavior using the Conners scale [194].

Lastly, on a microbial level, an RCT showed that the intake of PUFAs does not seem to affect the alpha or beta diversity of the microbiota of the participants. Nevertheless, it did show a reversible increase in genera, such as *Bifidobacterium roseburia* and *Lactobaccilus* spp., all of which are important for the production of SCFAs and maintain an anti-inflammatory environment [195]. Similarly, a commentary discussing the importance of long-chain PUFAs as a mean to restore a healthy gut microbiome, deduced that PUFA ingestion may act as a protector from developing systemic inflammation and in the long term, chronic disease. It is, therefore, hypothesized that PUFA supplementation would not only be of therapeutic importance for ADHD, but also a prophylactic measurement against cancer as inflammation leads to immunosuppression and activates immune checkpoints resulting in an optimal tumor microenvironment [196].

Although the collected data show inconclusive results concerning the effect of PUFA supplementation as a therapeutic measurement for ADHD, the indirect effects of ingesting PUFAs and its impact on the microbiome may as well be crucial determinants that could modify the metabolism and consequently the behavioral and cognitive symptoms of ADHD.

### 4.7. Antibiotics

Although the development of antibiotics has made it possible to treat life-threatening infections, the use of antibiotics reduces the microbiota diversity in the GI-tract [197]. Consequently, the use of antibiotics may elevate the number of pathogenic bacteria, such as *Enterobacter, Klebsiella, Citrobacter, and Pseudomonas* and decrease anaerobic bacteria [197]. For example, a human study analyzing the short term parenteral-neonatal antibiotic usage showed that it reduced the number of protective *Bifidobacteria* in the first couple of months of life [198]. Supporting these results, Penders et al. not only found a decrease of *Bifidobacteria*, but also of *Bacteroides* when infants administered antibiotics [122].

Results concerning the correlation of early antibiotic use and later risk of developing ADHD seem to be incoherent. A Danish population-based cohort study did not find an association in sibling-stratified Cox model between antibiotic use in the first two years of life and the risk of developing ADHD [130]. Another study, however, using 871 European newborns examined the effects of early antibiotic treatment on cognitive functions with the help of IQ and reading tests, and on symptoms of ADHD using the mentioned Conners Rating Scale-Revised (CRS-R). Thereby they discovered that children who consumed antibiotics in the first year of life showed a reduced reading ability score, higher scores on the CRS-R, rated by parents, and increased symptoms of ADHD at the ages of 7–11 years. Nonetheless, this association was not made for babies and children that used antibiotics between the ages of 12 months and 3.5 years. This indicates that one of the vital factors for developing ADHD is the age in which the newborn consumes the antibiotics. It seems that during the first 12 months of life, important developments of the gut-brain axis take place, which when disrupted influence the neurodevelopment, and thus, the CNS in the long run [12]. These data, however, must be interpreted with caution, as this was not an RCT. Accordingly, direct causation between the antibiotic use and later seen ADHD cannot be correctly made [199].

### 4.8. Probiotics

By definition of the FAO/WHO probiotics are ”live microorganisms which when administered in adequate amounts confer a health benefit on the host” [200]. Benefits of probiotics include reinforcing a more desirable environment in the gut, a healthy digestive system, and finally an adequate immune system [201]. Thereby probiotics help to sustain and produce healthy enzymes while eradicating potentially harmful pathogens [202,203]. Naturally occurring probiotic sources include lactic acid fermented vegetables, such as kimchi or fermented dairy products as, for example, yogurt [204].

The influence of probiotic strains on psychiatric diseases has been examined by multiple studies, concluding a positive effect on such illnesses and are, thus, described as “psychobiotics” [90]. An animal study using mice showed that probiotic ingestion of *Bifidobacterium longum* and *breve* led to a reduction of depression and anxiety symptoms [205].

The seminal study by Pärtty et al. researched the effects of probiotic use on the development of ADHD in children by randomly administering strains of *Lactobacillus rhamnosus* into 75 infants. The infants were monitored at three weeks, 3, 6, 12, 18 and 24 months, and finally, at 13 years of age. The authors concluded that at the age of 13 years, ADHD was diagnosed in 6/35 (17.1%) children using the placebo, whereas no children had this disorder in the probiotic group. These results, even if encouraging, do not identify any specific composition of the microbiota to the neurodevelopmental disease, and thus, might mean that probiotics decrease ADHD in a different way rather than influencing the composition of the microbiome [142]. However, it is important to conclude that these findings potentially represent a method to reduce the risk of developing ADHD.

## 5. Discussion

This literature review demonstrates that the ADHD population has a different gut microbial composition in comparison to healthy controls as the phylum Actinobacteria is more and Firmicutes less abundant in ADHD patients. The genus *Bifidobacterium*, belonging to the phylum Actinobacteria, seems to play a significant role in the pathogenesis of ADHD and is recurrently influenced by several factors. *Bifidobacteria* do not only protect the barrier function in the gut and support a healthy immune response [168], but also influence the dopamine system by elevating the production of CDT which increases phenylalanine levels, and finally, results in higher levels of dopamine. This review showed that *Bifidobacterium* was decreased in offspring that were born (i) via c-section delivery ([122,123], (ii) as preterms [144,206], (iii) were breastfed [164] or (iv) were given antibiotics in the first months of life [122,198]. All of these factors are simultaneously associated with an increased risk of developing ADHD. Nevertheless, using *Bifidobacterium* as a potential biomarker for diagnosis of ADHD seems uncertain due to varying results regarding *Bifidobacterium* levels in ADHD patients. Although Pärtty et al. observed decreased levels of *Bifidobacterium* in 3 and 6-month-old ADHD patients [142], Aarts et al. detected slightly increased levels of the genus using a larger sample size and a more sensitive methodology [69]. Thus, for future research, well-designed studies, using a larger sample size, are needed to deduce a definite correlation between levels of *Bifidobacterium* and ADHD and the importance of this genus as a biomarker.

Additionally, this article concludes that the concertation of neuroprotective BDNF, indirectly influenced by the microbiome [173], plays a vital role in the pathogenesis of ADHD. The majority of reports showed a negative correlation between levels of BDNF and ADHD [173,174,207]. As the levels of SCFAs [173], and PUFAs [185] are positively correlated with BDNF, omega-3 fatty acids may prove to be of therapeutic importance. So far, various studies have shown that adding PUFAs to the diet only marginally decreases the symptoms of ADHD [193,194,208]. Future studies could assess the effects of various concentrations of PUFAs and age at which these were ingested on the symptom development of ADHD. BDNF shows properties important for neurogenesis in the critical stages of neurodevelopment. The production of SCFAs by the microbiome has been positively associated with levels of BDNF [173]. Therefore, increasing SCFAs through fiber-rich nutrition in combination with the appropriate gut microbial composition could also be a beneficial means for the treatment of ADHD symptoms.

It is widely known that c-section delivery causes the offspring′s microbiome to be more similar to the mother′s skin rather than her vaginal flora. However, it is still under debate to what extent this change impacts the development of ADHD. We decided to concentrate on the more recent papers, that used a large sample size and a precise methodology by differentiating between elective and emergency c-sections. These studies show that not every c-section increases the risk of developing ADHD, but only those that were done intrapartum [129,130]. Although this correlation is most probably not due to a differing microbial composition and rather due to various confounders, such as gestational age and birth weight [130], it is still important to note that emergency c-sections bear an intrinsic risk for the offspring developing ADHD.

Additionally, it has become increasingly clear to what extent prematurity plays a role in the development of ADHD. As the GI-tract and its colonization with bacteria are still underdeveloped, the microbiome shows lower levels of neuroprotective *Lactobacillus* [144]. Nonetheless, this decrease of the genus has not yet been directly associated with the development of ADHD. Much more important seems to be the combination of premature infants having underdeveloped brain structures and an immature immune system resulting in being more prone to neuronal cell death and infections that promote neuroinflammation, and finally influence the neurodevelopment. It is difficult to deduce the exact impact of microbial changes in preterms on the development of ADHD, as there are numerous confounders [153]. Thus, future studies should elucidate and concentrate on levels of pro-inflammatory cytokines in neonates and determine the extent to which underdeveloped brain structures influence the development of ADHD. Once these have been thoroughly understood, one can assess in what way the microbiome plays a role in the pathophysiology of prenates having a higher prevalence of ADHD. As the topic of this literature review is relatively new, only a limited number of studies examining the link between ADHD and the microbiota could be found. Hence, it was challenging to draw concrete conclusions from the scarce available data. A solid conclusion will require future investigations enrolling larger populations with defined pathologies to be able to analyze the study outcomes using robust statistical analysis. Finally, it is important that future trials use standardized methodologies for an unambiguous comparison of the outcomes and results. This literature review has made it clear that certain factors are associated with ADHD, while simultaneously changing the guts microbiome. Nevertheless, it remains yet to be determined to what extent the composition of the microbiome in the gut influences the development of ADHD.

## 6. Conclusions

To determine to what extent the microbiome plays a role in the pathophysiology of ADHD, further studies are needed. We discussed several triggers that have been associated with ADHD, how these correlate with an altered microbial composition, and thus, how various microbes might act as possible biomarkers for ADHD. Further research, on the microbial composition of ADHD patients using large, well-diagnosed cohorts is needed in order to find future conclusive biomarkers and therapeutic methods to treat ADHD.

## Figures and Tables

**Table 1:** Various studies that tested phenylalanine levels in ADHD patients. ↑ represent the increase of phenylalanine found in ADHD patients and ↓ the decrease of the amino acid in comparison to healthy controls (HC). The symbol — describes that the study found no correlation between ADHD and phenylalanine levels. The accumulative data to date do not allow a definite correlation between a change in phenylalanine levels and ADHD. *p* levels less than 0.05 were considered statistically different.

Source	Levels of Phenylalanine in ADHD Patients	Sample Size (n)	Statistical Significance (*p*)
[69]	↑	96	*p* < 0.001
[73]	↑	79	*p* < 0.001
[74]	↓	44	*p* < 0.1
[75]	↓	48	*p* < 0.05
[76]	—	155	*p* < 0.01

**Table 2:** List of seven studies that tested the effects of c-section delivery on the development of ADHD. The table describes if the studies differentiated between the types of c-sections and their effects, and finally shows the sample size and statistical significance level of the individual studies. The symbol - represents that for these studies, this information could not be found as the studies were systematic reviews. The data shows that elective vs. emergency c-sections seem to have different effects on ADHD. *p* levels less than 0.05 were considered statistically different.

Source	Type of C-Section	Effect	Sample Size (n)	Statistical Significance (*p*)
[124]	No differentiation	Altered dopamine response	-	-
[126]	No differentiation	No effect	248	*p* = 0.005
[127]	No differentiation	No effect	12,991	*p* < 0.05
[128]	No differentiation	Positive correlation to ADHD	-	-
[129]	Elective vs. intrapartum	Only intrapartum c-sections showed a positive correlation to ADHD	1,722,548	*p* < 0.05
[130]	Elective vs. intrapartum	Only intrapartum c-sections showed a positive correlation to ADHD	671,592	*p* < 0.05
[131]	Elective vs. intrapartum	No effect	13,141	*p* < 0.05

**Table 3:** Listing the different genera, predominantly found in formula-fed vs. breastfed infants. The arrow ↑ describes that this genus is increased in variously fed infants, while ‘-‘ represents that there is no significant change in this genus. One can clearly see that microbial diversity is increased in formula-fed in comparison to breastfed infants. *p* levels less than 0.05 were considered statistically different.

Genus	Formula-Fed	Sample Size (n)	Statistical Significance (*p*)	Breastfed	Sample Size (n)	Statistical Significance (*p*)
*Bifidobacterium*	↑ [122]	232	*p* < 0.01	↑ [122]	700	*p* < 0.01
	↑ [164]	6	*p* <0.05	↑ [164]	6	*p* < 0.05
	↑ [165]	182	*p* < 0.001	↑ [165]	312	*p* < 0.001
*Escherichia coli*	↑ [122]	232	*p* < 0.01	-	700	*p* < 0.01
*Bacteroides*	↑ [122]	232	*p* < 0.01	-	700	*p* < 0.01
*Lactobacillus*	↑ [122]	232	*p* < 0.01	-	700	*p* < 0.01
*Veillonella parvula*	↑ [164]	6	*p* < 0.05	-	6	*p* < 0.05
*Streptococcus*	↑ [164]	6	*p* < 0.05	-	6	*p* < 0.05
*Clostridium coccoides*	↑ [165]	182	*p* < 0.014	-	312	*p* < 0.014
